# High-quality reconstruction of arrhythmic cardiac cycles

**DOI:** 10.1186/1532-429X-18-S1-P6

**Published:** 2016-01-27

**Authors:** Stefan Wundrak, Jan Paul, Michael Radermacher, Vinzenz Hombach, Wolfgang Rottbauer, Peter Bernhardt, Volker Rasche

**Affiliations:** grid.6582.90000000419369748Ulm University, Ulm, Germany

## Background

The current clinical standard in cardiac functional MRI is retrospectively gated cine MRI. The technique is relying on a regular heart beat and cardiac cycles showing substantial deviation from the mean heart rate are excluded, often causing reduced image quality and completely suppressing arrhythmic cycles. We introduce a new correlation based self-gating approach enabling reconstruction of the entire cardiac motion including arrhythmic sections at high image quality.

## Methods

### Study Population and MRI protocol

Dynamic short axis time-resolved cardiac data sets were acquired at 1.5T from three patients (2 women, 1 man, aged 79 to 83 years) with known severe cardiac arrhythmia resulting in varying cardiac cycle length even during a short 6.3 second breathhold. All data were obtained with a 32-element cardiac coil applying a tiny golden angle (Ψ_7_≈23.628°) [1] radial balanced SSFP sequence with TR / TE = 2.6 / 1.3 ms, flip angle = 60°, resolution 1.7^2^ mm^2^, slice thickness 8 mm, and acquisition matrix 212^2^.

### Reconstruction

The data were reconstructed applying the proposed non-uniform image-based self-gating technique (nuSG [2]), real-time compressed sensing reconstruction (GRASP [3]), and classical retrospective cardiac self-gating (SG). M-mode data were calculated from the short-axis images for better appreciation of arrhythmic cycles.

### Analysis

The visibility of the arrhythmic cycles was assessed in the M-mode data. Furthermore, end-systolic and end-diastolic endocardial areas and image sharpness was compared between the investigated reconstruction techniques.

## Results

**Figure 1:**
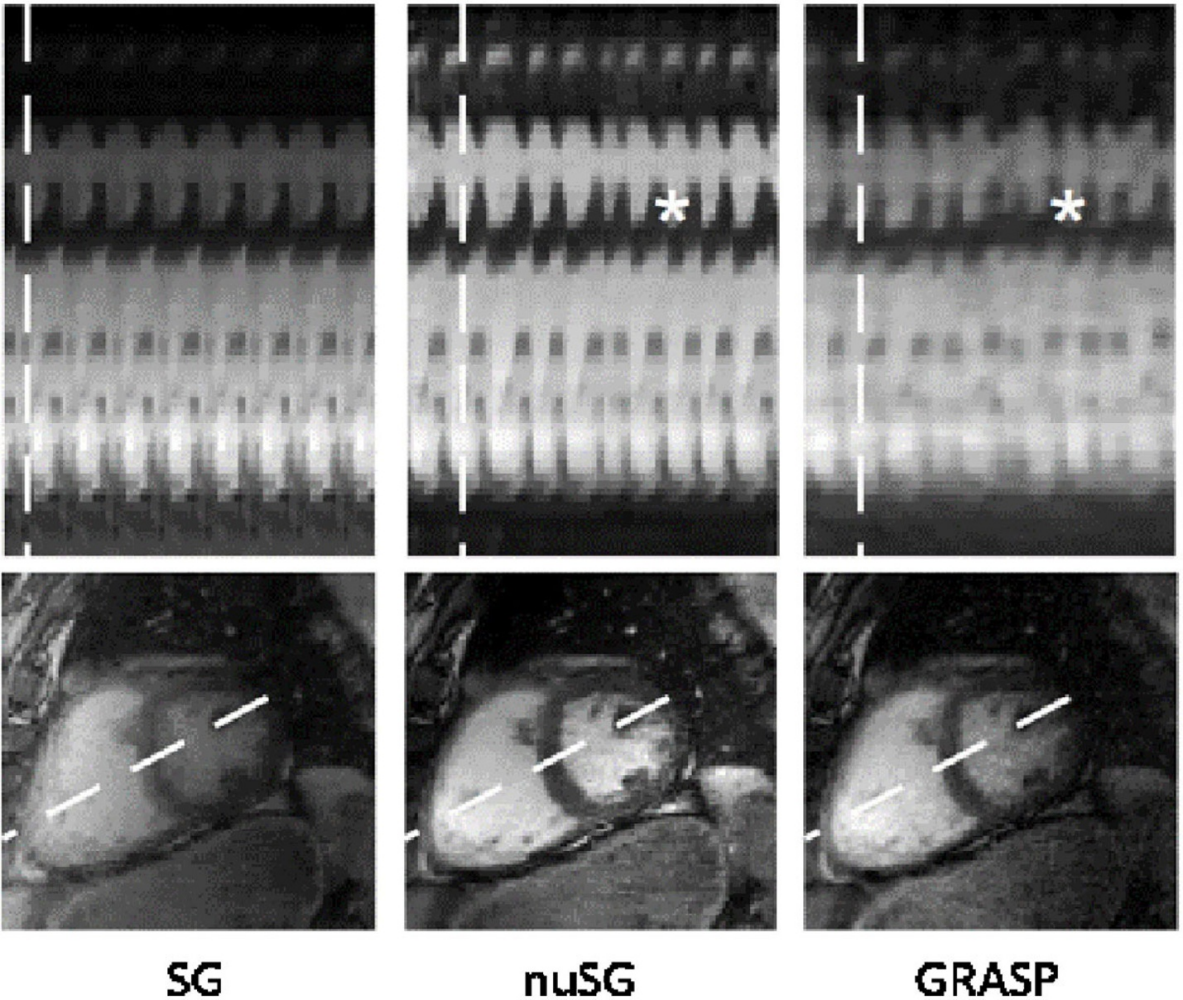
**Reconstructions obtained from conventional retrospective self-gating (SG), realtime CS (GRASP), and the proposed non-uniform self-gating (nuSG)**. Asterisks in the M-mode indicate arrhythmic cycles only visible with GRASP and nuSG.

Figure 1 shows exemplarily the resulting images and M-modes for one patient. Arrhythmic cycles can be well appreciated in the M-mode images obtained from the nuSG and GRASP reconstructions. Visually, the images from SG have more residual streaking artifacts and appear less sharp than the images from nuSG and the nuSG shows superior SNR compared to GRASP. nuSG resulted in an average improvement of the wall sharpness of up to 96%.. Endocardial areas (ED [ml]/ES [ml]) resulted as 15.7 ± 2.3 / 7.7 ± 2.5 (SG), 15.6 ± 1.1 / 8.4 ± 1.1 (nuSG), and 15.7 ± 2.1 / 11 ± 1.3 (GRASP).

## Conclusions

In conclusion, a new self-gating method was proposed that allows CMR of arrhythmic patients at high image quality, maintaining the information of the arrhythmic cycles in the resulting images. Compared to real-time techniques, the nuSG technique provides higher SNR and image sharpness.
